# Trimethyllysine, a trimethylamine N-oxide precursor, predicts the presence, severity, and prognosis of heart failure

**DOI:** 10.3389/fcvm.2022.907997

**Published:** 2022-09-29

**Authors:** Xiao Zong, Qin Fan, Qian Yang, Roubai Pan, Lingfang Zhuang, Rui Xi, Ruiyan Zhang, Rong Tao

**Affiliations:** ^1^Department of Cardiovascular Medicine, Ruijin Hospital, Shanghai Jiao Tong University School of Medicine, Shanghai, China; ^2^Shanghai Jiao Tong University School of Medicine, Institution of Cardiovascular Diseases, Shanghai, China

**Keywords:** trimethyllysine, heart failure, prognosis, gut microbiota, metabolite

## Abstract

**Background and aims:**

Intestinal flora metabolites are associated with cardiovascular (CV) diseases including heart failure (HF). The carnitine precursor trimethyllysine (TML), which participates in the generation of the atherogenic-related metabolite trimethylamine N-oxide (TMAO), was found to be related to poor prognosis in patients with CV diseases. The aim of the present study was to examine the relationship between TML and stable chronic HF.

**Methods and results:**

In total, 956 subjects including 471 stable chronic HF and 485 non-HF patients were enrolled in the present cohort study and subjects with stable HF were followed up for 2.0 ± 1.1 years. Serum levels of TML and TMAO were measured by liquid chromatography mass spectrometry in tandem. TML levels were significantly elevated in patients with HF compared with non-HF patients and were positively correlated with N-terminal pro-brain natriuretic peptide (NTproBNP) levels (*r* = 0.448, *P* < 0.001). TML was associated with the presence of HF after adjusting for age, sex, complications, traditional clinical factors, and TMAO (tertile 3 (T3), adjusted odds ratio (OR) 1.93, 95% confidence interval (CI) 1.19–3.13, and *P* = 0.007). In patients with HF, increased TML levels were associated with a composite endpoint of CV death and HF hospitalization during follow-up (T3, adjusted hazard ratio (HR) 1.93, 95% CI 1.27–2.93, and *P* = 0.002). Increased TML levels indicated a higher risk of CV death, re-hospitalization, and all-cause mortality.

**Conclusion:**

Serum TML levels were associated with the presence and severity of HF in all subjects. High levels of TML can indicate complications and poor prognosis in HF patients.

## Introduction

Heart failure (HF) is a complex clinical syndrome that results from any structural or functional impairment of ventricular filling or ejection of blood (1). Intestinal flora can serve as an endocrine organ that contributes to the pathogenesis of HF through its metabolites (2–4). In particular, trimethylamine N-oxide (TMAO), which is an intestine-derived metabolite of the choline/carnitine metabolic pathway, has been proven to be a metabolite potentially linking intestinal flora to cardiovascular (CV) disease (5, 6). Recent studies have also found that increased TMAO levels can predict poor prognosis in patients with both acute (6) and chronic HF (7, 8).

However, nowadays the exploration between intestinal flora metabolites and HF mainly concentrated on TMAO. Within the TMAO-related intestinal flora pathway, other metabolites also showed significant association with CV risk and CV diseases, including trimethyllysine (TML) (9, 10).

The amino acid TML, which is introduced by various animal- and plant-derived dietary sources, can work as a precursor for intestinal flora-dependent generation of TMAO (11) as well as a regulator of carnitine biosynthesis (12). Since increased TML levels were determined to be related to higher CV risk in subjects who were taken cardiac risk evaluation (11), several studies have probed into the association between TML and CV diseases. TML was found to be related to an increased risk of mortality in patients with evidence of carotid artery atherosclerosis in one small study (9). It is associated with the progression of atherosclerosis (13) and is involved a variety of different pathways potentially leading to atherogenesis (14). TML could also independently predict the risk of acute myocardial infarction among patients with suspected angina pectoris (15), and predict adverse CV prognosis among patients presenting with acute coronary syndrome (10). Recent research found a relationship between TML and the risk of mortality in subjects with acute HF (16); however, at present, whether serum TML levels are associated with disease severity in stable chronic HF has not been well discovered.

Therefore, the present study was to examine the relationship between systemic TML levels and the presence as well as prognosis of patients with stable chronic HF.

## Materials and methods

### Study population

The present study was a prospective cohort study designed to investigate the intestinal microbiota metabolite levels in patients with HF. A total of 956 including 471 stable chronic HF and 485 non-HF patients hospitalized in the Department of Cardiovascular Medicine, Ruijin Hospital, were included consecutively. Patients with severe concomitant diseases, such as infections, connective tissue diseases, autoimmune diseases, malignancies, and gastrointestinal disorders were excluded. Patients with acute myocardial infarction and cerebrovascular events during the last 6 months were also excluded. However, no information was available from patients with the probability of excessive intake of antioxidants, vitamins, micronutrients, or fish oils. The study was conducted in accordance with the Declaration of Helsinki and was approved by the Ethics Committee of Ruijin Hospital, Ethics Committee reference number: 2016-019. All patients were explained about the study and provided informed consent before enrollment.

### Follow-up and outcomes

The present study included the primary outcome and three secondary outcomes. A composite endpoint including CV death and first re-hospitalization because of HF was defined as the primary outcome. The secondary outcomes consisted of CV death, hospitalization due to HF, and all-cause mortality. Follow-up survey was conducted by hospital visits or through telephone. All endpoints were confirmed by independent cardiologists intensively. Recurrent HF was diagnosed on account of signs and symptoms, abnormal laboratory parameters, as well as imageological examination.

### Detection of trimethyllysine and trimethylamine N-oxide

Fasting serum blood samples were collected and frozen at –80^°^C until analysis to avoid oxidation or modification. None of the participants performed any exercise prior to blood collection. Levels of TML and TMAO in serum were detected by liquid chromatography-mass spectrometry (LC-MS). Isotopically labeled TML-d9 (sc-475693, Santa Cruz Biotechnology, Santa Cruz, CA, USA) and TMAO-d9 (sc-475042, Santa Cruz Biotechnology, Santa Cruz, CA, USA) were used as internal standards. 150 μl of TML-d9 (20 μg/ml, dissolved in ultrapure water) and 500 μl of TMAO-d9 (1 ug/ml, dissolved in ultrapure water) were added to 200 ml of pure methanol, then vortexed and mixed to obtain the internal standard dilution working solution (ISWS). 50 μl of serum sample was placed in a 96-well plate, and 400 μl of ISWS was added to each sample, vortexed and mixed for 15 min, followed by centrifugation at 3,000 g for 15 min at 4^°^C. 150 μl supernatant was then transferred to the 96-well plate and tested on the machine.

Samples were separated on a 1.7 μm (2.1 × 50 mm) column (ACQUITY UPLC BEH Amide, Waters, Milford, MA, USA) through the high-performance liquid chromatography (UltiMate 3000, Dionex, Sunnyvale, CA, USA) using an elution gradient of two mobile phases (A) 10 mM ammonium acetate 0.1% formic acid and (B) 10 mM ammonium acetate 95% acetonitrile 0.1% formic acid. Quantification was performed with a triple quadrupole mass spectrometry (TSQ Vantage, Thermo Scientific, Waltham, MA, USA) with positive electrospray ionization in the multiple reaction monitoring mode where the precursor ion (parent ion) of the target substance was first screened by the quadrupole and the fragment ions of the precursor ion were filtered through QQQ to select a desired characteristic fragment ion for quantification. The retention times of TML and TMAO were 2.54 and 1.5 min, respectively. The MRM-transitions monitored were for TML m/z 189 > 84, for TML-d9 m/z 198 > 84, for TMAO m/z 76 > 58, and for TMAO-d9 m/z 85 > 66. The peak area of each analyte was corrected by the peak area of the internal standards compound, and the concentrations of TML and TMAO in the serum sample were calculated semi-quantitatively by the ratio of the peak area of each analyte to the internal standards compound, and further quantified according to the standard concentration.

The same volume of serum was drawn from each sample, pooled and mixed, which is the quality control (QC) sample. During the testing process, every 12 samples were followed by a QC sample. The samples and the machine can be considered stable if the relative standard deviation (RSD) of QC samples were below 15%. And the RSD of TMAO and TML in the QC sample was 6.51 and 3.71%, respectively.

### Echocardiographic measurements

Echocardiography was conducted by experienced cardiac sonographers and analyzed by independent cardiologists. M-mode echocardiography was used to assess left atrial diameter (LAD), left ventricular end diastolic diameter (LVEDD), and left ventricular end systolic diameter (LVESD). And the biplanar Simpson’s method was performed to determine the cardiac function as measured by left ventricular ejection fraction (LVEF).

### Statistical analyses

Continuous variables were shown as mean ± standard deviation (SD), and expressed per SD for regression. Log transformation was performed before analysis if it was not normally distributed. The comparison between two groups was conducted by unpaired *t*-test, while trends over tertiles were tested by one-way ANOVA analysis. Categorical variables are presented as counts (%) and significant differences between groups were determined using the chi-square test or Fisher’s exact test.

Simple correlation analyses were performed to investigate the relationship between TML and different clinical features. Logistic regression models were used to determine the association between TML and the presence of HF. Odds ratios (ORs) with 95% CIs for HF were obtained for both univariable and multivariable analyses. The adjusted model 1 consisted of both age and sex. The full adjustment model 2 contained age, sex, body mass index (BMI), hypertension, diabetes mellitus (DM), hemoglobin, albumin, creatinine, glycosylated hemoglobin, low-density lipoprotein cholesterol, high-sensitivity C-reactive protein (hsCRP), and TMAO.

For follow-up data, the relationship between TML (as shown by tertiles) and the primary endpoint and secondary endpoints were visualized using Kaplan–Meier plots and assessed by log-rank tests. Cox regression models in univariate, model 1 (age and sex) adjusted, and model 2 adjusted analyses were performed to calculate hazard ratios (HRs) and 95% CIs. In regression analyses, results were presented according to per 1 SD increment of log-transformed TML concentrations and the 3rd versus 1st tertile. Patients who were lost during follow-up were analyzed according to the censored data. Statistical analyses were performed using SPSS version 22.0 software, and the two-sided *P*-value < 0.05 was defined as statistically significant.

## Results

### Baseline characteristics

A total of 956 subjects participated in the present study including 471 stable chronic HF and 485 non-HF patients. In all subjects, the average age was 61.4 ± 10.9 years, 62.8% were men, 55.4% had hypertension, 25.1% had DM, 15.4% had dyslipidemia, and 16.0% had renal dysfunction.

Baseline demographic, clinical, and laboratory characteristics of all participants was shown in Table 1 (grouped by TML tertile) and Supplementary Table 1 (grouped by HF or non-HF). Patients in the higher TML level group were more possible to have complicating diseases such as hypertension, DM, and renal dysfunction. There was a positive association between incremental TML levels and the increase in HbA1c, creatinine, uric acid, hsCRP, and N-terminal pro-brain natriuretic peptide (NTproBNP). Patients with higher TML levels had lower albumin levels as well as estimated glomerular filtration rates (eGFR). TML serum concentrations were also significantly correlated with TMAO levels (*r* = 0.395, *P* < 0.001).

**TABLE 1:** Baseline characteristics of all subjects.

	TML < 0.54 μM (*n* = 318)	0.54 μM ≤ TML < 0.75 μM (*n* = 319)	TML ≥ 0.75 μM (*n* = 319)	*P*-value
**Demographic characteristics**				
Age (years)	60.1 ± 9.5	61.3 ± 11.7	62.7 ± 11.4	0.014
Male	135 (42.5)	226 (70.8)	239 (74.9)	<0.001
Current smoking	65 (20.4)	115 (36.1)	134 (42.0)	<0.001
Current drinking	57 (17.9)	77 (24.1)	77 (24.1)	0.092
Body mass index (kg/m^2^)	24.4 ± 3.3	25.2 ± 3.7	24.9 ± 3.8	0.015
Systolic blood pressure (mmHg)	132.3 ± 18.7	130.3 ± 20.2	131.1 ± 20.6	0.435
Diastolic blood pressure (mmHg)	76.6 ± 11.4	76.0 ± 13.4	76.2 ± 13.2	0.802
Heart rate (beats/min)	78.7 ± 11.2	79.9 ± 13.4	79.8 ± 15.2	0.438
Family history	37 (11.6)	50 (15.7)	51 (16.0)	0.219
**Medical history**				
Hypertension	150 (47.2)	177 (55.5)	203 (63.6)	<0.001
Diabetes mellitus	58 (18.2)	88 (27.6)	94 (29.5)	0.002
Dyslipidemia	47 (14.8)	50 (15.7)	50 (15.7)	0.937
Renal dysfunction	8 (2.5)	44 (13.8)	101 (31.7)	<0.001
Stroke	24 (7.5)	16 (5.0)	44 (13.8)	<0.001
**Lab. examination**				
WBC (*10^9/L)	6.1 ± 1.9	6.5 ± 2.0	6.7 ± 2.0	0.001
Hemoglobin (g/L)	135.7 ± 13.8	138.3 ± 15.7	135.7 ± 20.3	0.069
Platelet (*10^9/L)	191.5 ± 52.9	182.7 ± 50.6	181.5 ± 54.9	0.034
HbA1c (%)	6.0 ± 1.0	6.2 ± 1.1	6.3 ± 1.1	0.005
ALT (IU/L)	30.5 ± 86.1	33.0 ± 55.6	31.5 ± 40.7	0.883
Albumin (g/L)	39.6 ± 3.6	38.6 ± 3.7	38.0 ± 4.7	<0.001
Creatinine (μmol/L)	69.6 ± 14.2	82.2 ± 27.3	118.3 ± 120.5	<0.001
Uric acid (μmol/L)	322.7 ± 90.4	373.4 ± 110.2	403.3 ± 124.9	<0.001
eGFR (ml/min/1.73 m^2^)	88.2 ± 16.5	81.3 ± 18.8	68.9 ± 24.5	<0.001
Triglyceride (mmol/L)	1.5 ± 0.9	1.6 ± 1.0	1.6 ± 1.3	0.341
Total cholesterol (mmol/L)	4.4 ± 1.8	4.1 ± 1.1	4.1 ± 1.5	0.021
LDL-C (mmol/L)	2.5 ± 0.9	2.5 ± 1.0	2.4 ± 0.9	0.207
HDL-C (mmol/L)	1.2 ± 0.3	1.1 ± 0.3	1.1 ± 0.3	<0.001
Tropnin I (ng/ml)	0.6 ± 4.2	1.0 ± 6.3	1.5 ± 8.5	0.228
hsCRP (mg/L)	3.7 ± 11.4	5.6 ± 16.5	10.2 ± 28.3	0.001
NTproBNP (pg/ml)	528.8 ± 1232.8	1358.7 ± 3282.8	3732.3 ± 7777.7	<0.001
D-dimer (mg/L)	0.5 ± 1.4	0.6 ± 1.4	0.7 ± 1.1	0.161
TMAO (μM)	0.9 ± 1.0	1.2 ± 1.5	2.2 ± 4.1	<0.001
LAD (mm)	37.8 ± 6.2	40.9 ± 6.8	42.9 ± 7.0	<0.001
LVEDD (mm)	50.6 ± 8.1	54.9 ± 9.8	58.0 ± 9.5	<0.001
LVESD (mm)	34.5 ± 10.3	39.9 ± 12.0	43.6 ± 12.1	<0.001
LVEF (%)	59.1 ± 14.8	51.9 ± 16.7	46.4 ± 16.4	<0.001
**Medications**				
ACEI/ARB/ARNI	128 (40.3)	186 (58.3)	212 (66.5)	<0.001
β-blocker	169 (53.1)	207 (64.9)	224 (70.2)	<0.001
Spironolactone	42 (13.2)	94 (29.5)	132 (41.4)	<0.001
Statins	224 (70.4)	248 (77.7)	239 (74.9)	0.104
Hypoglycemic drugs	47 (14.8)	69 (21.6)	69 (21.6)	0.041

ACEI, angiotensin-converting enzyme inhibitor; ALT, alanine transaminase; ARB, angiotensin receptor blocker; ARNI, angiotensin receptor enkephalin inhibitor; eGFR, estimated glomerular filtration rates; HbA1c, glycosylated hemoglobin; HDL-C, high-density lipoprotein cholesterol; hsCRP, high sensitivity C reactive protein; LAD, left atrial diameter; LDL-C, low-density lipoprotein cholesterol; LVEDD, left ventricular end diastolic diameter; LVEF, left ventricular ejection fraction; LVESD, left ventricular end systolic diameter; NTproBNP, N-terminal pro-brain natriuretic peptide; TMAO, trimethylamino oxide; TML, trimethyllysine; WBC, white blood cells. *Means multiply.

### Trimethyllysine levels were associated with the presence and severity of heart failure

**FIGURE 1:**
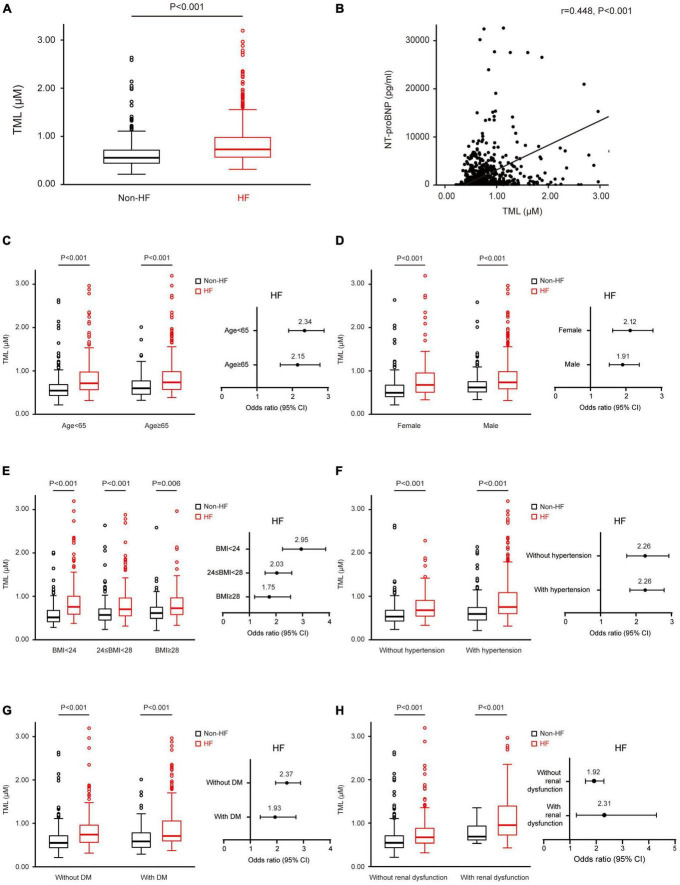
Trimethyllysine (TML) levels are associated with the presence and severity of heart failure (HF) in all subjects and different subgroups. **(A)** TML levels are increased in patients with HF compared with the non-HF group in all subjects at baseline. **(B)** Simple correlation analysis between serum levels of TML and N-terminal pro-brain natriuretic peptide (NTproBNP). Box–Whisker plots of TML levels among patients with and without HF in different subgroups, including ages <65 and ≥65 **(C)**; male and female **(D)**; normal, overweight, and obesity **(E)**; with and without hypertension **(F)**; with and without DM **(G)**; as well as with and without dyslipidemia **(H)**. Forest plots illustrate the odds of the presence of HF according to TML levels in these subgroups **(C–G)**. Symbols represent odds ratios (ORs) and the 95% confidence intervals are shown by line length.

Initial examination of the distribution of TML levels showed remarkably higher TML levels (0.9 ± 0.5 vs. 0.6 ± 0.3 μM, *P* < 0.001) among subjects with HF (Figure 1A). TML concentrations were significantly correlated to NTproBNP levels (*r* = 0.448, *P* < 0.001, Figure 1B), LAD (*r* = 0.322, *P* < 0.001), LVEDD (*r* = 0.361, *P* < 0.001), and LVESD (*r* = 0.323, *P* < 0.001), and negatively associated with LVEF (*r* = –0.324, *P* < 0.001).

Patients belonging to the highest tertile of TML concentrations had a significantly higher risk of HF compared with participants in the lowest tertile (Table 2). After adjusting for age, sex, BMI, hypertension, DM, hemoglobin, albumin, creatinine, LDL, HbA1c, hsCRP, and TMAO, increased TML levels remained associated with significantly increased odds for HF (tertile 3 (T3) vs. tertile 1, OR: 1.93, 95% CI: 1.19–3.13, *P* = 0.007). As a continuous variable, TML levels could also predict the presence of HF at baseline after full adjustment (per 1 SD, OR: 1.28, 95% CI: 1.02–1.61, *P* = 0.037) (Table 2). The receiver operating characteristic (ROC) curve of TML in predicting HF was 0.71 (0.67–0.74, *P* < 0.01), and the predictive cut-off value of TML was 0.7 μM.

**TABLE 2:** Trimethyllysine (TML) was associated with the presence of heart failure (HF) in all subjects.

	Unadjusted OR	*P*-value	Adjusted for model 1 OR	*P*-value	Adjusted for model 2 OR	*P*-value
log TML per SD	2.31 (1.96–2.72)	<0.001	1.93 (1.63–2.29)	<0.001	1.28 (1.02–1.61)	0.037
TML tertiles	2.32 (1.96–2.75)	<0.001	1.88 (1.56–2.27)	<0.001	1.39 (1.10–1.77)	0.007
Tertile 1	1 (ref)		1 (ref)		1 (ref)	
Tertile 2	2.57 (1.86–3.57)	<0.001	1.68 (1.17–2.42)	0.005	1.25 (0.79–1.96)	0.344
Tertile 3	5.38 (3.84–7.56)	<0.001	3.53 (2.44–5.11)	<0.001	1.93 (1.19–3.13)	0.007

OR, odds ratio; SD, standard deviation; TML, trimethyllysine. Model 1: adjusted for age and sex. Model 2: adjusted for age, sex, body mass index (BMI), hypertension, diabetes mellitus (DM), hemoglobin, albumin, creatinine, low-density lipoprotein cholesterol, glycosylated hemoglobin, high sensitivity C reactive protein (hsCRP), and trimethylamino oxide.

### Trimethyllysine levels could predict the presence of heart failure in different subgroups

We also analyzed age- and sex-specific associations of circulating TML concentrations with the presence of HF. In both patients aged <65 and ≥65 years, those with HF had higher levels of TML, and TML could predict HF in both groups (Figure 1C). Increased TML levels were correlated with elevated odds of HF more remarkably in women than in men (Figure 1D). TML was more significantly correlated with HF in subjects with normal weight than in those who were overweight or obese (Figure 1E). Moreover, subgroup analyses demonstrated that increased TML levels could predict the presence of HF among patients with or without a history of hypertension, DM, and renal dysfunction, respectively, more remarkably in those without DM and in those with renal dysfunction (Figures 1F–H).

### Trimethyllysine levels were associated with the complications and poor prognosis in patients with heart failure

**FIGURE 2:**
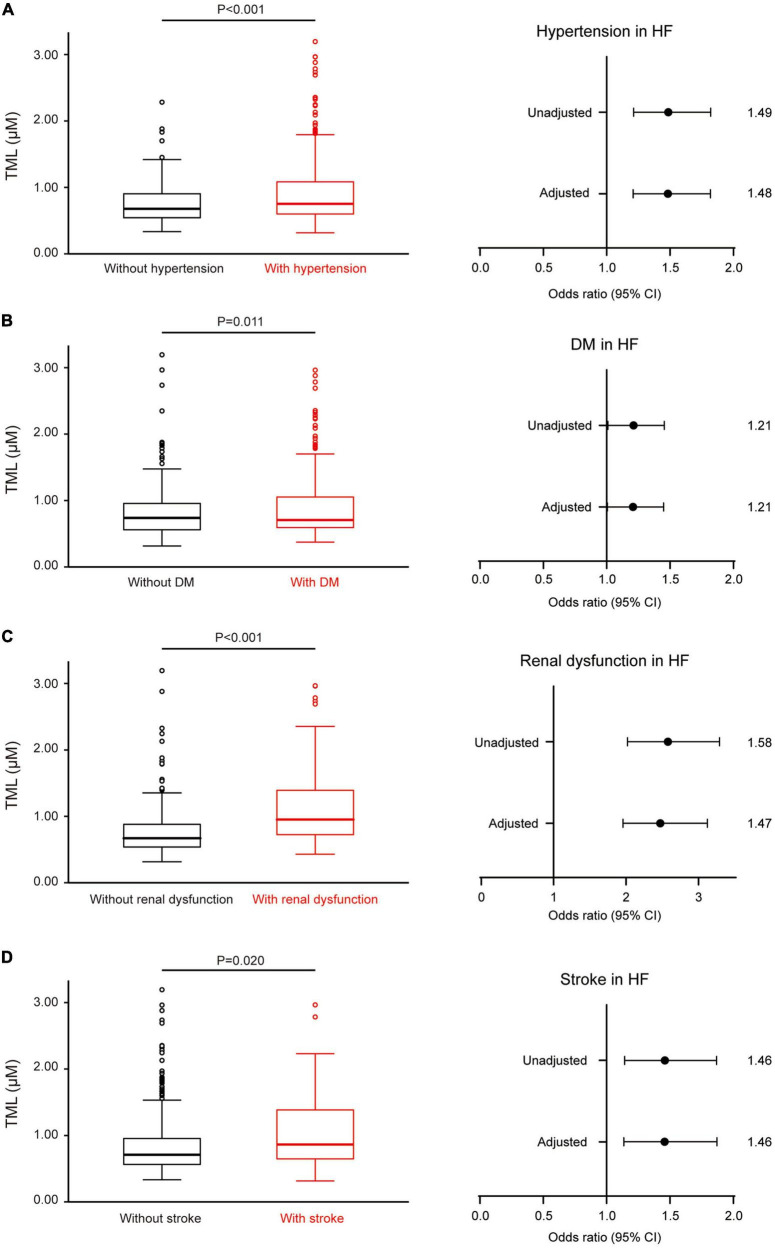
Trimethyllysine (TML) levels are associated with several complications in patients with heart failure (HF). **(A)** In HF patients, serum levels of TML are elevated in those with hypertension than those without it. The forest plot at right illustrates the odds of the presence of hypertension according to TML levels in patients with HF, both unadjusted and adjusted for age, sex, and body mass index (BMI). Similar results were found regarding diabetes mellitus (DM) **(B)**, renal dysfunction **(C)**, and stroke **(D)**.

Among all subjects, 471 were diagnosed with stable systolic HF. TML levels were higher among those with hypertension, DM, renal dysfunction, and stroke in the HF patients. TML was also associated with the presence of these complications after adjustment (Figure 2).

During a mean follow-up of 2.0 ± 1.1 years, outcomes consisted of all-cause mortality in 66 patients (14.0%) including CV death in 57 patients (12.1%), re-admission because of HF in 111 patients (23.6%), and the composite primary endpoint in 154 patients (32.7%). Therefore, we first investigated the association between TML levels and primary as well as secondary outcomes in patients with HF.

**FIGURE 3:**
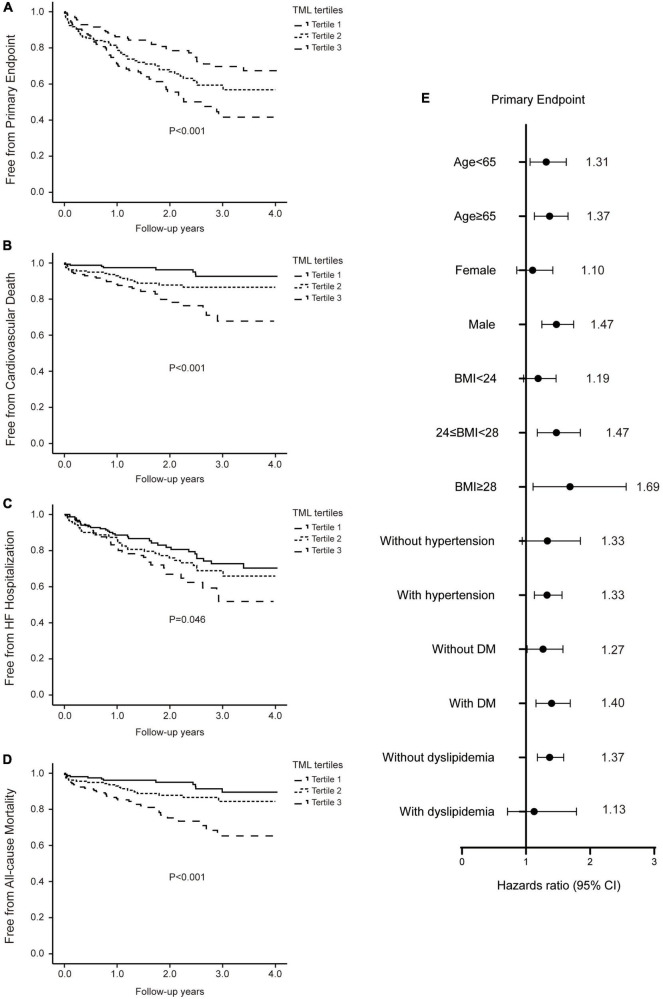
Increased trimethyllysine (TML) levels are associated with poor prognosis in patients with heart failure (HF). Kaplan–Meier curves and log-rank analyses illustrating the risks of the primary endpoint **(A)**, cardiovascular (CV) death **(B)**, hospitalization due to HF **(C)**, and all-cause mortality **(D)**, stratified by the tertiles of TML levels in patients with HF. **(E)** Forest plots indicating the risks of the primary endpoint in different subgroups according to TML levels in patients with HF. Symbols represent hazard ratios (HRs) and line length indicates the 95% confidence intervals.

Patients who experienced the primary endpoint were more likely to have higher serum TML levels at presentation (1.0 ± 0.7 vs. 0.8 ± 0.4 μM, *P* = 0.004). Kaplan–Meier analyses and survival curves illustrated enhanced rates of the primary endpoint with elevating TML levels (log-rank *P* < 0.001, Figure 3A). Similarly, patients in the higher TML level group had a higher risk of experiencing CV death, HF re-admission, and all-cause mortality during follow-up (Figures 3B–D).

Following multivariable cox regression analyses adjusting for conventional risk factors including age, sex, BMI, hypertension, DM, hemoglobin, albumin, creatinine, LDL, HbA1c, and TMAO, elevated (tertile 3 vs. tertile 1) serum TML remained independently correlated to increased risk of the primary endpoint (HR: 1.93, 95% CI: 1.27–2.93, *P* = 0.002) (Table 3). Furthermore, patients in the highest tertile of TML levels demonstrated a significantly elevated risk of CV death (HR: 3.96, 95% CI: 1.80–8.72, *P* = 0.001), HF hospitalization (HR: 1.75, 95% CI: 1.09–2.80, *P* = 0.019), and all-cause mortality (HR: 3.34, 95% CI: 1.68–6.64, *P* = 0.001), compared with those in the lowest tertile after full adjustment (Table 3). TML concentrations could also predict the primary endpoint in HF patients belonging to different subgroups (Figure 3E).

**TABLE 3:** Trimethyllysine (TML) was associated with poor prognosis in patients with heart failure (HF).

	Unadjusted HR	*P*-value	Adjusted for model 1 HR	*P*-value	Adjusted for model 2 HR	*P*-value
**Primary endpoint**						
log TML per SD	1.35 (1.17–1.55)	<0.001	1.33 (1.16–1.53)	<0.001	1.20 (1.03–1.39)	0.018
TML tertiles	1.49 (1.22–1.82)	<0.001	1.51 (1.24–1.85)	<0.001	1.37 (1.12–1.68)	0.002
Tertile 1	1 (ref)		1 (ref)		1 (ref)	
Tertile 2	1.60 (1.05–2.44)	0.03	1.66 (1.09–2.54)	0.019	1.62 (1.06–2.47)	0.026
Tertile 3	2.25 (1.49–3.39)	<0.001	2.33 (1.54–3.52)	<0.001	1.93 (1.27–2.93)	0.002
**Cardiovascular death**						
log TML per SD	1.66 (1.34–2.06)	<0.001	1.64 (1.32–2.02)	<0.001	1.41 (1.13–1.76)	0.002
TML tertiles	2.10 (1.47–3.01)	<0.001	2.15 (1.50–3.08)	<0.001	1.94 (1.35–2.78)	<0.001
Tertile 1	1 (ref)		1 (ref)		1 (ref)	
Tertile 2	2.27 (0.99–5.23)	0.053	2.212 (0.96–5.12)	0.064	2.24 (0.97–5.16)	0.059
Tertile 3	4.56 (2.09–9.96)	<0.001	4.67 (2.13–10.21)	<0.001	3.96 (1.80–8.72)	0.001
**HF hospitalization**						
log TML per SD	1.22 (1.02–1.45)	0.028	1.21 (1.02–1.44)	0.028	1.17 (0.99–1.40)	0.073
TML tertiles	1.34 (1.06–1.69)	0.014	1.36 (1.08–1.71)	0.01	1.32 (1.05–1.67)	0.019
Tertile 1	1 (ref)		1 (ref)		1 (ref)	
Tertile 2	1.31 (0.81–2.11)	0.267	1.36 (0.84–2.19)	0.212	1.33 (0.82–2.15)	0.243
Tertile 3	1.79 (1.12–2.85)	0.015	1.84 (1.16–2.94)	0.01	1.75 (1.09–2.80)	0.019
**All-cause mortality**						
log TML per SD	1.62 (1.32–1.98)	<0.001	1.59 (1.30–1.94)	<0.001	1.41 (1.14–1.73)	0.002
TML tertiles	2.02 (1.45–2.80)	<0.001	2.05 (1.47–2.86)	< 0.001	1.85 (1.33–2.57)	<0.001
Tertile 1	1 (ref)		1 (ref)		1 (ref)	
Tertile 2	1.77 (0.84–3.71)	0.134	1.71 (0.81–3.61)	0.162	1.73 (0.82–3.64)	0.150
Tertile 3	3.88 (1.97–7.66)	<0.001	3.96 (2.00–7.82)	<0.001	3.34 (1.68–6.64)	0.001

Model 1: adjusted for age and sex. Model 2: adjusted for age, sex, body mass index (BMI), hypertension, diabetes mellitus (DM), hemoglobin, albumin, creatinine, low-density lipoprotein cholesterol, glycosylated hemoglobin, and trimethylamino oxide.

## Discussion

**FIGURE 4:**
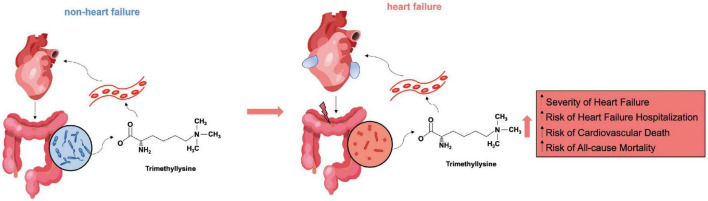
Summary diagram outlining the proposed relationship between trimethyllysine (TML) and heart failure (HF). In non-HF conditions, TML synthesis and blood entry are minimal under the protection of the balanced intestinal flora and a healthy intestinal barrier. When HF occurs, the heart’s ability to pump blood decreases, the gut becomes congested and edematous, and the prevalent harmful bacteria, as well as the impaired intestinal barrier together, make TML easier to enter the bloodstream. Elevated circulating levels of TML are independently associated with the severity and poor prognosis of HF.

The present study probed into the potential impact of TML on the presence and prognosis of HF. The major findings are as follows: First, patients with HF had significantly increased serum levels of TML compared with those without HF. Second, higher serum TML concentrations are independently associated with HF. Higher TML levels could also significantly predict the complications and poor prognosis in patients with HF, including CV death and HF re-admission. The proposed interaction between TML and HF was summarized in Figure 4. These results indicate that detection of serum TML may provide further value for making out patients at risk for HF and long-term poor prognosis.

A healthy intestinal barrier prevents intestinal bacteria and harmful substances from entering the bloodstream. As a result of HF, the intestine becomes congested and edematous, the intestinal barrier is damaged, and intestinal flora and metabolites enter the bloodstream, aggravating inflammation and cardiac dysfunction (4). Many intestinal flora metabolites are associated with HF, particularly TMAO (7, 17). A study analyzing some TMAO-carnitine/choline pathway metabolic products found a relationship between TML and poor prognosis in patients with acute HF (16). Another small study investigating carnitine metabolism and HF also detected increased levels of L-carnitine and its precursor TML in patients with HF (18). TML levels were positively related to NTproBNP and CRP levels and negatively associated with cardiac index and eGFR (18), which was in accordance with the results of the present study. Nonetheless, it is the first study to indicate the predictive value of TML in patients with stable HF with a relatively large population.

In spite of the discovered association between TML and incident HF risks as well as poor prognosis, the underlying mechanisms interpreting the strong relationship have not been fully elucidated. On the one hand, a recent study found that trimethyl-5-aminovaleric acid, a derivative of TML, inhibits fatty acid metabolism in cardiac myocytes and exacerbates myocardial hypertrophy, and HF (19), which could be a possible way in which TML plays a role in HF. On the other hand, TML is also an intermediate, rate-limiting factor for carnitine biosynthesis (20), and carnitine may play a role in HF pathophysiology. Carnitine is an important transporter to carry fatty acid into mitochondria (21, 22). However, there is a shift in energy metabolism in the pathological heart during HF, indicated by the reduced utilization of fatty acids and the increased utilization of glucose and ketone bodies (23). As a probably compensatory mechanism to rescue fatty acid metabolism, thus improving cardiac function, circulating carnitine levels are found to increase during HF (16, 18). Thus, our observations suggest that measuring TML levels, as an intermediate in carnitine biosynthesis, may discover patients with severe cardiac metabolic dysfunction, thereby causing HF, and poor prognosis. The current study also found that TML was correlated with hsCRP, which indicates that TML may also play a part in the inflammatory process during HF.

Another mechanism is the potential function of TML to form TMA and TMAO, the prothrombotic and atherogenic metabolite (11). Gut microbiota-derived metabolite TMAO plasma levels have been found to be positively correlated to increased CV risk and mortality (24). It can modulate cholesterol and sterol metabolism in multiple compartments and enhance atherosclerosis (25). Previous studies have also found a relationship between TML and atherosclerosis (9, 13). In patients with acute coronary syndrome, TML is a predictor of future major adverse CV events (10, 11). However, elevated TAMO was not associated with cardiac systolic dysfunction, although high TMAO was observed in HF patients and portended long-term mortality risk (7), while our study found a relationship between TML and systolic dysfunction. The present study also investigated the relationship between TML and TMAO levels. In line with recent studies (10, 11, 15), a strong association between serum levels of TML and TMAO was observed at baseline. However, different from other TMAO-producing dietary sources, such as carnitine, choline, or betaine, whose prognostic value was abolished following the inclusion of TMAO into regression models (26, 27), TML could predict the presence of HF in all subjects as well as the poor prognosis in HF patients independent of TMAO. Thus, alternative mechanisms to TMAO may also explain the observed relationship between TML and HF, which needs to be explored in future studies.

On the other hand, the major sources of TML in the human body involve both exogenous and endogenous mechanisms. Exogenous assumptions from both animal-and plant-based foods, such as meat, seafood, and vegetables, are an important dietary source of TML, where TML is widely present in the protein-bound form (11, 28, 29). Endogenous production of TML is a portion of the post-translational modification of proteins in mammals. It took part in the process of the modification in histone during chromatin remodeling as well as gene expression regulation (30–32). The identification of the contributions of microbial versus eukaryotic cells in TML production will have an important impact in exploring the function of TML in CV diseases including HF.

In the present study, we indicated a significant relationship between TML and the complications of HF, including hypertension, DM, renal dysfunction, and stroke. At baseline, elevated TML levels were discovered among participants with a medical history of hypertension, DM, or renal dysfunction, which is in accordance with the findings of other cohorts (15). Another study also demonstrated that in patients with coronary artery disease, serum TML levels can predict the risk of type 2 DM independently of traditional risk factors (33), partly due to dysfunctional acid metabolism. Although the specific mechanisms underlying each complication need to be explored in future studies, these complications, which are related to increased TML levels, could further influence the overall state of patients with HF and lead to a high mortality rate and poor prognosis.

Among all these processes, which one is the major underlying mechanism accounting for the association between serum TML levels and HF has not been identified, and it is debated whether increased TML concentration is the cause or result of HF, which needs further exploration. Nevertheless, TML may help identify patients with a high risk of HF and poor prognosis, and promotion in preventive risk-reducing efforts in HF.

### Study limitations

First, this was a single-center study, and data about dietary intake which could affect metabolite levels were not available. The present study also did not specifically differentiate the etiologies of HF and compared TML levels in each group. Furthermore, our study measured circulating TML levels at a single time point, and the changes in TML levels as time goes on need to be examined in future studies. Some unidentified confounding effects might not have been evaluated, and the study was not designed to detect other carnitine/choline metabolites besides TMAO. The mechanisms underlying the relationship between TML and HF will be explored in further studies. Nonetheless, our study provides novel insights which provide clinical links between intestinal flora-associated metabolite TML and the pathophysiology of HF with application prospects and research value.

## Conclusion

In conclusion, serum levels of TML provide independent clinical value for the presence and severity of chronic HF. It could also provide clinical value for poor prognosis and mortality among patients with chronic HF.

## Data availability statement

The raw data supporting the conclusions of this article will be made available by the authors, without undue reservation.

## Ethics statement

The studies involving human participants were reviewed and approved by the Ethics Committee of Ruijin Hospital, Ethics Committee reference number: 2016-019. The patients/participants provided their written informed consent to participate in this study.

## Author contributions

XZ, QF, QY, and RT contributed to all stages including the conception and design of this study and interpretation of the data. XZ, RP, QY, and LZ were integral to the design of the study and the data collection. QF and RX took charge of the statistical analysis. RX and RZ also assisted with the interpretation of the data. QF contributed to drafting the article, together with RZ and RT participating in revising the version to be submitted. All authors assisted with preparing the manuscript itself as well as revising the manuscript for important intellectual content, and they also had read and approved the manuscript.

## Supplementary material

Supplementary Figure 1 Receiver operating characteristic (ROC) curve for predicting the presence of heart failure (HF).
