# Supplementary material for: Trimethyllysine, a trimethylamine N-oxide precursor, predicts the presence, severity, and prognosis of heart failure
Source: Front Cardiovasc Med. 2022 Sep 29;9:907997. doi: 10.3389/fcvm.2022.907997 (PMC9558138; doi:10.3389/fcvm.2022.907997)
Supplement: Supplementary file 1 [file Table_1.docx]

**Supplemental Table 1. Baseline characteristics of all subjects.**

|  | Non-HF  (n=485) | | Chornic HF  (n=471) | P value |
| --- | --- | --- | --- | --- |
| *Demographic characteristics* | |  |  |  |
| Age (years) | 60.2±9.7 | | 62.5±12.0 | <0.01 |
| Male | 207 (42.7) | | 393 (83.4) | <0.01 |
| Current Smoking | 95 (19.6) | | 219 (46.5) | <0.01 |
| Current Drinking | 75 (15.5) | | 136 (28.9) | <0.01 |
| Body mass index (kg/m^2^) | 24.8±3.4 | | 24.9±3.8 | 0.52 |
| Systolic blood pressure (mmHg) | 135.0±18.1 | | 127.4±20.8 | <0.01 |
| Diastolic blood pressure (mmHg) | 77.0±11.0 | | 75.5±14.2 | 0.08 |
| Heart rate (beats/min) | 77.9±10.8 | | 81.1±15.5 | <0.01 |
| Family history | 53 (10.9) | | 85 (18.0) | <0.01 |
| *Medical history* |  | |  |  |
| Hypertension | 239 (49.3) | | 291 (61.8) | <0.01 |
| Diabetes mellitus | 61 (12.6) | | 179 (38.0) | <0.01 |
| Dyslipidemia | 77 (15.9) | | 70 (14.9) | 0.66 |
| Renal dysfunction | 20 (4.1) | | 133 (28.2) | <0.01 |
| Stroke | 26 (5.4) | | 58 (12.3) | <0.01 |
| *Lab. Examination* |  | |  |  |
| WBC (*10^9/L) | 5.9±1.7 | | 6.9±2.2 | <0.01 |
| Hemoglobin (g/L) | 136.7±13.1 | | 136.4±20.0 | 0.80 |
| Platelet (*10^9/L) | 189.1±47.7 | | 181.3±50.7 | 0.02 |
| HbA1c (%) | 5.7±0.9 | | 6.5±1.9 | <0.01 |
| ALT (IU/L) | 22.9±15.9 | | 40.6±82.3 | <0.01 |
| Albumin (g/L) | 39.7±3.1 | | 37.7±4.7 | <0.01 |
| Creatinine (μmol/L) | 71.1±15.2 | | 109.6±101.7 | <0.01 |
| Uric acid (μmol/L) | 321.7±81.0 | | 412.7±124.8 | <0.01 |
| eGFR (ml/min/1.73m^2^) | 86.3±16.3 | | 72.4±24.3 | <0.01 |
| Triglyceride (mmol/L) | 1.5±0.9 | | 1.6±1.3 | 0.50 |
| Total cholesterol (mmol/L) | 4.4±1.6 | | 3.9±1.4 | <0.01 |
| LDL-C (mmol/L) | 2.6±0.9 | | 2.4±0.9 | <0.01 |
| HDL-C (mmol/L) | 1.3±0.3 | | 1.1±0.3 | <0.01 |
| Tropnin I (ng/ml) | 0.2±2.5 | | 1.9±9.0 | <0.01 |
| hsCRP (mg/L) | 2.6±10.5 | | 10.8±26.5 | <0.01 |
| NTproBNP (pg/ml) | 85.9±239.9 | | 3716.6±6799.5 | <0.01 |
| D-dimer (mg/L) | 0.3±0.5 | | 0.9±1.7 | <0.01 |
| TMAO (μM) | 1.1±1.5 | | 1.8±3.4 | <0.01 |
| TML (μM) | 0.6±0.3 | | 0.9±0.5 | <0.01 |
| LAD (mm) | 36.2±3.8 | | 45.0±6.7 | <0.01 |
| LVEDD (mm) | 47.3±3.7 | | 62.0±8.1 | <0.01 |
| LVESD (mm) | 29.3±2.9 | | 49.7±8.8 | <0.01 |
| LVEF (%) | 67.7±4.0 | | 36.8±8.6 | <0.01 |
| *Medications* |  | |  |  |
| ACEI/ARB/ARNI | 148 (30.5) | | 378 (80.3) | <0.01 |
| β-blocker | 187 (38.6) | | 413 (87.7) | <0.01 |
| Spironolactone | 2 (0.4) | | 266 (56.5) | <0.01 |
| Statins | 328 (67.6) | | 383(81.3) | <0.01 |
| Hypoglycemic drugs | 44 (9.1) | | 141 (29.9) | <0.01 |

ACEI, angiotensin-converting enzyme inhibitor; ALT, alanine transaminase; ARB, angiotensin receptor blocker; ARNI, angiotensin receptor enkephalin inhibitor; eGFR, estimated glomerular filtration rates; HbA1c, glycosylated hemoglobin; HDL-C, high-density lipoprotein cholesterol; hsCRP, high sensitivity C reactive protein; LAD, left atrial diameter; LDL-C, low-density lipoprotein cholesterol; LVEDD, left ventricular end diastolic diameter; LVEF, left ventricular ejection fraction; LVESD, left ventricular end systolic diameter; NTproBNP, N-terminal pro-brain natriuretic peptide; TMAO,trimethylamino oxide; TML, trimethyllysine; WBC, white blood cells

* means multiply.
