# Supplementary figures and images for: Trimethyllysine, a trimethylamine N-oxide precursor, predicts the presence, severity, and prognosis of heart failure
Source: Front Cardiovasc Med. 2022 Sep 29;9:907997. doi: 10.3389/fcvm.2022.907997 (PMC9558138; doi:10.3389/fcvm.2022.907997)

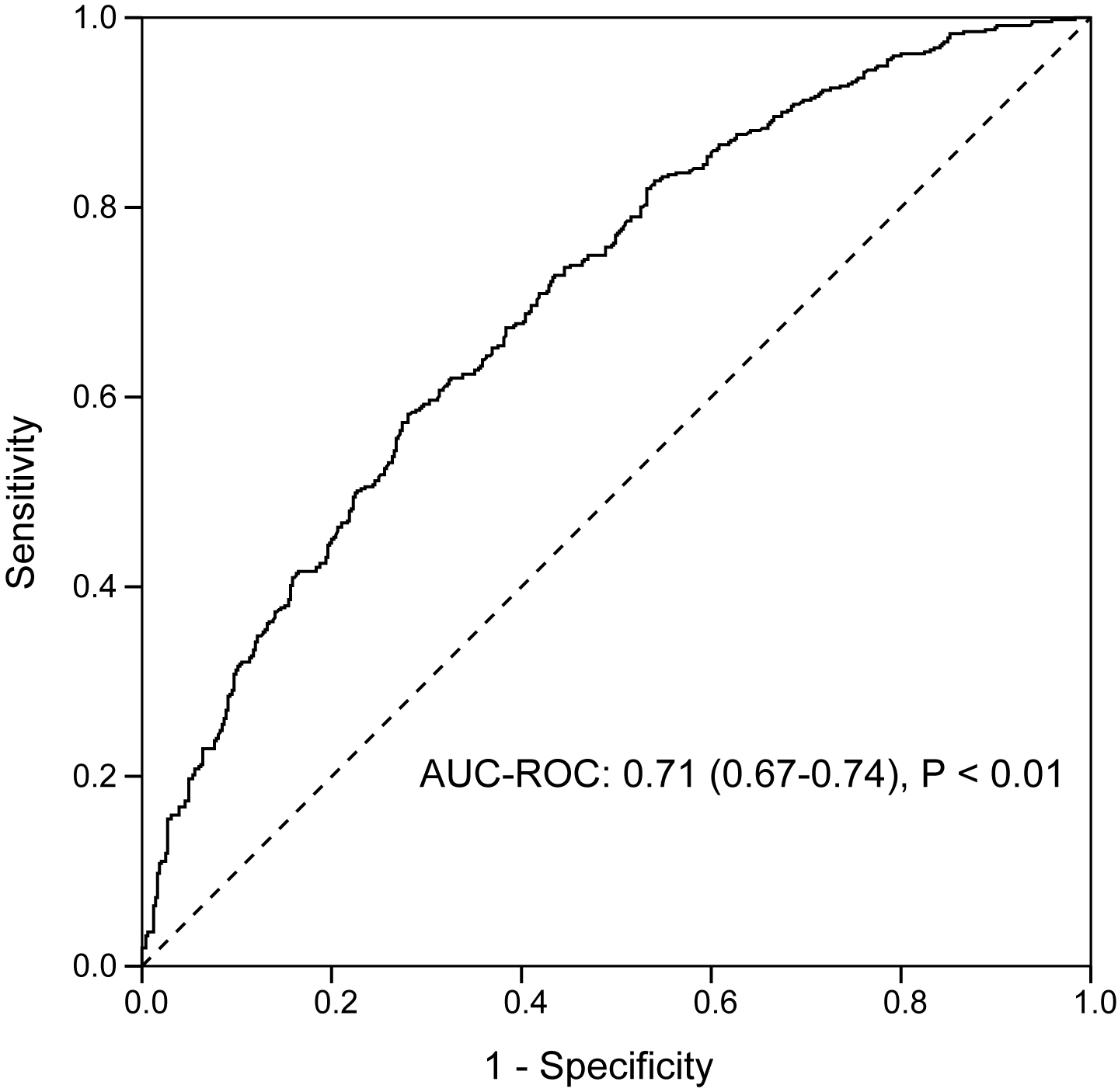

Supplement: Supplementary Figure 1 — Receiver operating characteristic (ROC) curve for predicting the presence of heart failure (HF). [file Image_1.TIF]
